# Bisphosphonate Treatment in a Patient Affected by MPS IVA with Osteoporotic Phenotype

**DOI:** 10.1155/2013/891596

**Published:** 2013-11-18

**Authors:** Albina Tummolo, Orazio Gabrielli, Alberto Gaeta, Maristella Masciopinto, Lucia Zampini, Luigi Michele Pavone, Paola Di Natale, Francesco Papadia

**Affiliations:** ^1^Metabolic Diseases and Clinical Genetics Unit, Children's Hospital Giovanni XXIII, Via Amendola 207, 70126 Bari, Italy; ^2^Department of Clinical Sciences, Polytechnic University of Marche, Azienda Ospedali Riuniti, Via Conca 71, 60126 Ancona, Italy; ^3^Radiology Unit, Children's Hospital Giovanni XXIII, Via Amendola 207, 70126 Bari, Italy; ^4^Department of Molecular Medicine and Medical Biotechnologies, University of Naples Federico II, Zona Ospedaliera, Via Pansini 5, 80131 Naples, Italy

## Abstract

Morquio A syndrome (Mucopolysaccharidosis type IVA) (MPS IVA) is a rare inherited metabolic disorder characterized by the defective degradation of keratan sulfate and chondroitin-6-sulfate. Classically, MPS IVA patients present with severe multisystemic involvement and have a short life expectancy. Attenuated forms with clinical features limited to minor skeletal abnormalities and short stature have also been described, sometimes associated to an early-onset osteoporotic phenotype. No treatment with allogenic bone marrow transplantation or gene therapy is currently available for Morquio A syndrome, and enzyme replacement therapy is under evaluation. We report a case of MPS IVA, who manifested tardily attenuated phenotype and significant bone mass reduction, which was treated with a bisphosphonate (BPN), resulting in an improvement of X-ray skeletal aspects and functional bone performance. We suggest that the use of bisphosphonates may be an interesting supportive therapeutic option for Morquio A patients with osteoporotic phenotype, but further studies involving more patients are necessary to confirm our findings.

## 1. Introduction

Morquio A syndrome (Mucopolysaccharidosis type IVA) (MPS IVA) is a rare inherited metabolic disorder characterized by the defective degradation of keratan sulfate and chondroitin-6-sulfate, due to the abnormal or absent activity of N-acetylgalactosamine-6-sulfatase (GALNS gene) [1].

 Classically, Morquio A patients do not present mental retardation, but usually manifest significant skeletal abnormalities such as pectus carinatum, rib flaring, genua valga, and dysostosis multiplex. Spine involvement is one of the most recognizable skeletal aspects with platyspondyly, kyphosis, gibbus, and hypoplasia or the absence of odontoid process, leading to atlantoaxial instability and cervical myelopathy. Joint ligament laxity is also detectable and is associated with frequent dislocations (hips and knees) and joint abnormalities. Corneal opacities and deafness are also features of the syndrome [2]. 

Different phenotypes of MPS IVA have been recognized. The most severe phenotypes are characterized by severe pulmonary involvement, quadriparesis, and death, usually between the second and third decade of life. In attenuated forms, a longer life expectancy has been observed [3], and clinical features may be limited to minor skeletal abnormalities and short stature [4]. In some cases, the diagnosis may be delayed as the urinary glycosaminoglycans (GAGs) may be in the normal range. 

No treatment with allogenic bone marrow transplantation or gene therapy is currently available for Morquio A syndrome, and enzyme replacement therapy is under evaluation. Palliative measures include surgery for skeletal abnormalities and multispecialist support for systemic involvement. 

In the last few years, MPS IVA has been reported to be associated to an early-onset osteoporotic phenotype, which can affect the clinical course of this condition. The accumulation of keratan sulfate disturbing bone mass acquisition and perturbing the regular microarchitecture of bone tissue has been reported to reflect on bone mass density (BMD) in this condition [5].

We report a case of MPS IVA, who presented with attenuated phenotype and significant bone density reduction, which was treated with a bisphosphonate (BPN), resulting in the improvement of X-ray skeletal aspects and functional bone performance.

## 2. Case Report

The patient is the second son of nonconsanguineous Italian parents. He was born with full-term uneventful delivery. Growth rate and psychomotor development were normal during the neonatal period and early childhood.

The patient was referred to our hospital at the age of 11 because of pain in the lower limbs and concomitant functional limitation. 

On examination, he showed short stature, although compatible with the parents' height, mild facial dysmorphism with normal psychomotor development, and normal visual and hearing ability. 

**Figure 1:**
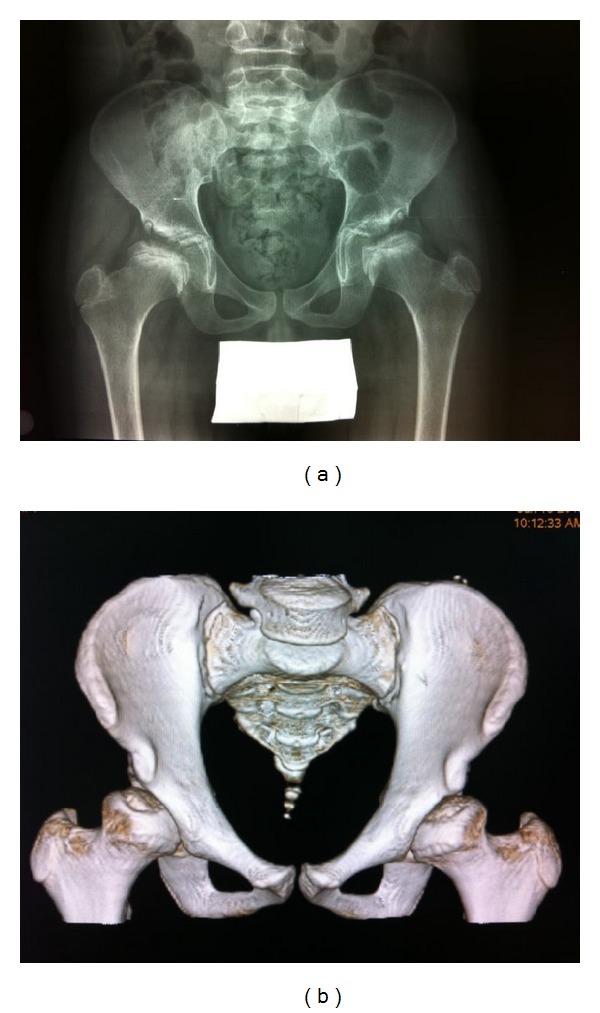
(a) First pelvic X-ray: dysplastic appearance of both femoral heads and irregular upper contour of the acetabular roof with mild coxa vara. (b) Last pelvic CT scan (three-dimensional volume-rendered reformatted picture): almost total reconstruction of anatomic structures.

The radiological evaluation showed accentuated dorsal kyphosis and lumbar lordosis, dysplastic appearance of both femoral heads, and irregular upper contour of the acetabular roof (Figure 1(a)). The abnormal aspect of dorsolumbar metamers was also detected, but there no dysplastic appearance of the odontoid process.

Pelvic Computed Tomography (CT) scan highlighted a hypoplasic aspect of femoral cephalic nuclei growth and an irregular delineation of the acetabular articular surfaces and cephalic polar profiles. The radiological finding of “coxa vara” led to the radiological diagnosis of bilateral Perthes' disease. 

Nonsteroidal anti-inflammatory drugs (NSAIDs) were therefore commenced. However, after a three-week treatment protocol, no significant improvement was observed. On the contrary, the patient manifested a gradual and steady deterioration of the initial conditions, resulting in a complete functional limitation of the lower limbs.

The study of the bone metabolism showed normal bone alkaline phosphatase, 84.5 *μ*g/L (n.v. 24–89), and osteocalcin 54.4 ng/mL (n.v. 24–123), and abnormal serum biomarkers, (vitamin D: 6 ng/mL (n.v. 13–67)). However, dual-energy X-ray absorptiometry (DXA) showed a severe reduction in bone mineral content, in the range of severe osteoporosis: total BMD of 0.456 g/cm^2^, 56%, *Z*-score −3.65.

The systemic osteoarthropathy and mild facial coarsening prompted further investigation for lysosomal storage diseases.

Quantitative evaluation of urinary mucopolysaccharides [6] was normal (12 *μ*g/mg creatinine, normal range 10–63), but electrophoresis of urinary mucopolysaccharides revealed a band corresponding to keratan sulfate. The enzymatic assay [7] revealed a decreased activity of galactose-6-sulfate sulfatase: 0.9 nmol/17h/mg protein (normal values 48.8+/−17.0, range 19.5–77.2). 

The diagnosis of MPS type IVA was confirmed by molecular analysis of the GALNS gene, which detected a compound heterozygosity of genotype p.H154Q/p.G290S (base change c.518C>A/c.924G>A), consistent with the diagnosis of Morquio A syndrome. The known polymorphism p.G290S was also found. 

In view of the reduced mobility and the significant reduction in the bone mineral content, we decided to commence a therapeutic protocol with a BPN namely, neridronate, given at a dose of 2 mg/kg, infused intravenously in two hours every 3 months [8].

The patient was followed up for the duration of therapy with clinical, radiological, and biochemical monitoring, during which we observed a gradual improvement in posture and mobility as well as a gradual and steady reduction in joint pain. 

After two years of therapy, the bone turnover indexes were all found to be in the normal range (vitamin D: 18.1 ng/mL, bone alkaline phosphatase: 38 *μ*g/L, and osteocalcine: 80.5 ng/mL) and the mean lumbar spine (L1–L4) BMD was 0.85 g/cm^2^, *Z*-score: +0.17.

CT scan showed an almost total reconstruction of anatomic structures. In particular, the acetabular roof presented greater regularity, although a few small geodic cavities could be detected at the proximal femoral epiphysis, which still appeared slightly flattened (Figure 1(b)). 

At ten months from the end of the treatment with the BPN, no adverse effects have been detected. The patient can walk normally, his height percentile has improved, and he is able to perform sports activity. 

## 3. Discussion

Morquio A syndrome is a rare condition, and in Italy its estimated incidence is about 1 : 1250000 live births [9], although the ongoing newborn screening strategies will probably alter these data in the near future. This condition is characterized by different degrees of severity of the clinical picture, which may result in a delayed diagnosis.

The diagnosis is usually made between the ages of 2 and 6, when skeletal abnormalities become more evident and cause not only retarded and altered ambulation but also severe growth retardation.

Our patient manifested in late childhood mild bone abnormalities and osteoporosis but no other peculiar features of MPS IVA.

Hecht et al. [10] reported a case of a 14-year-old boy with an attenuated form of Morquio syndrome who presented as having severe bilateral Perthes' disease. Other cases have been described [4, 11, 12], thus suggesting that the attenuated form of MPS IVA may not be so rare as was thought and its features may be misdiagnosed.

In our patient, the residual enzyme activity was 1.84% of the wild-type activity. DNA analysis resulted in the heterozygous phenotype p.H154Q/p.G290S. Mutation p.G290S was first described in a British patient with a severe form [13] and was subsequently observed in other severe cases. The other mutation, p.H154Q, has not been reported previously; it was not found in 100 normal alleles, thus suggesting that it may be a novel mutation. Since this alteration occurred in a nonconserved amino acid of the GALNS protein, it probably explains the 1.84% residual enzyme activity and probably accounts for the attenuated phenotype in our patient. The effect of attenuated mutations in MPS IVA patients was reported by Montaño et al. [14]: mutants found in the severe phenotypes had less than 1% of normal activity, while most mutants found in the attenuated phenotype had a higher residual activity (2.2–36% of the wild-type activity).

The radiological study, particularly with the integration of traditional radiology and CT scan, prompted us to suspect the clinical diagnosis and was also important for the follow up.

The main differential diagnosis of MPS IV remains bilateral Legg-Perthes' disease due to avascular injury of the femoral head. This condition, however, is usually unilateral and shows significant improvement after a few weeks of treatment with NSAIDS.

MPS IVA has already been associated to early-onset osteoporosis [15]. Other studies found an association between other forms of MPS and the development of early osteoporosis [16, 17]. Several possible causes have been suggested, among which were the lack of adequate nutrients (calcium and vitamins) and the lack of exposure to sunlight. An interesting possible explanation may come from studies by Li et al. [18], who first highlighted how the accumulation of keratan and chondroitin-6-sulfate, differently from other glycosaminoglycans (GAGs), determines excessive collagenolytic activity of a protease highly expressed in osteoclasts and involved in bone reabsorption: cathepsin K. The promotion of the collagenolytic activity of this degrading protease in MPS IVA may be involved in the pathogenesis of the osteoporotic phenotype recognizable in some patients. 

To our knowledge, this is the first case of Morquio A syndrome treated with a BPN.

BPNs are widely prescribed for osteoporosis, both in adults and in children, showing significant efficacy in increasing bone mass and preventing fractures in several randomized controlled trials [19]. Once incorporated into newly formed bone, BPNs can persist there for years through multiple cycles of bone reabsorption and deposition [20]. They inhibit bone resorption by impairing several osteoclast activities necessary for it and by promoting osteoclast apoptosis [21]. The main indication in childhood is for osteogenesis imperfecta, but they are also used in other forms of primary and secondary childhood osteoporosis. The most serious side effects of BPN use, such as uveitis, thrombocytopenia, and avascular necrosis of the jaw, have rarely been reported in children and adolescents [22].

In our experience, neridronate given intravenously to pediatric patients was not associated to significant adverse events.

In conclusion, mild skeletal abnormalities may be the only initial signs of attenuated forms of MPS IVA. Bone mass density deficiency may be detectable and can greatly worsen the clinical course of these patients. 

The use of BPNs may result in morphological as well as functional improvements in the bone and may be an interesting supportive therapy for Morquio A patients with osteoporotic phenotype, but further studies involving more patients are needed to confirm our findings.

## Abbreviations

MPSIVA: Mucopolysaccharidosis type IVA
GALNS: N-Acetylgalactosamine-6-sulfatase
GAGs: Glycosaminoglycans
BPN: Bisphosphonate
CT: Computed tomography
BMD: Bone mass density
NSAIDs: Nonsteroidal anti-inflammatory drugs
DXA: Dual-Energy X-ray absorptiometry.
